# The Semantic Content of Abstract Concepts: A Property Listing Study of 296 Abstract Words

**DOI:** 10.3389/fpsyg.2018.01748

**Published:** 2018-09-19

**Authors:** Marcel Harpaintner, Natalie M. Trumpp, Markus Kiefer

**Affiliations:** Department of Psychiatry, Ulm University, Ulm, Germany

**Keywords:** abstract concepts, grounded cognition, hierarchical cluster analysis, embodiment, semantic memory, conceptual representation, embodied cognition, language comprehension

## Abstract

The relation of abstract concepts to the modality-specific systems is discussed controversially. According to classical approaches, the semantic content of abstract concepts can only be coded by amodal or verbal-symbolic representations distinct from the sensory and motor systems, because abstract concepts lack a clear physical referent. Grounded cognition theories, in contrast, propose that abstract concepts do not depend only on the verbal system, but also on a variety of modal systems involving perception, action, emotion and internal states. In order to contribute to this debate, we investigated the semantic content of abstract concepts using a property generation task. Participants were asked to generate properties for 296 abstract concepts, which are relevant for constituting their meaning. These properties were categorized by a coding-scheme making a classification into modality-specific and verbal contents possible. Words were additionally rated with regard to concreteness/abstractness and familiarity. To identify possible subgroups of abstract concepts with distinct profiles of generated features, hierarchical cluster analyses were conducted. Participants generated a substantial proportion of introspective, affective, social, sensory and motor-related properties, in addition to verbal associations. Cluster analyses revealed different subcategories of abstract concepts, which can be characterized by the dominance of certain conceptual features. The present results are therefore compatible with grounded cognition theories, which emphasize the importance of linguistic, social, introspective and affective experiential information for the representation of abstract concepts. Our findings also indicate that abstract concepts are highly heterogeneous requiring the investigation of well-specified subcategories of abstract concepts, for instance as revealed by the present cluster analyses. The present study could thus guide future behavioral or imaging work further elucidating the representation of abstract concepts.

## Introduction

Research on conceptual knowledge is an important topic in cognitive psychology and in cognitive science in general. Central human abilities, such as problem solving, action planning, object recognition, communication and language crucially depend on conceptual knowledge stored in semantic long-term memory (Tulving, 1972; Humphreys et al., 1988; Kiefer and Pulvermüller, 2012). There is an agreement that concepts are the basic units of cognition. Concepts are defined as mental entities, which provide factual knowledge by integrating our sensory and motor experiences with the environment in a categorical fashion (Humphreys et al., 1988; Kiefer and Pulvermüller, 2012). They refer to concrete objects, but also to referents, which are not directly observable, like mental or emotional states, abstract ideas, social constellations and scientific theories. A main question in this regard concerns the role of modality-specific systems in the representation of conceptual knowledge.

Traditional models of semantic memory propose that concepts are represented in an amodal, symbolic format distinct from sensory and motor systems (Collins and Loftus, 1975; Anderson, 1978; Pylyshyn, 1980; Fodor, 2001; Mahon and Caramazza, 2009). These models have the advantage to naturally explain the representation of all concepts since an amodal and symbolic code is very potent in terms of computational power (Rogers et al., 2004; Patterson et al., 2007). This becomes especially evident when considering abstract concepts, which lack a clear physical referent by definition. At a first glance, abstract concepts do not rely on sensory and motor or other modality-specific information so that their representation might be quite naturally explained within an amodal theoretical framework. At the neural level, anterior (Patterson et al., 2007; Visser et al., 2010) and posterior (Gold et al., 2006; Hoffman et al., 2012) temporal cortices have been proposed as the central correlates of conceptual representation, serving as amodal semantic hubs. Such amodal theories assume that sensory and motor brain systems are not causally involved in retrieving conceptual information, their engagement is rather seen as an epiphenomenon (McClelland and Rogers, 2003).

More recent embodied or grounded cognition theories (Gallese and Lakoff, 2005; Pulvermüller, 2005; Barsalou, 2008; Borghi and Cimatti, 2009; Kousta et al., 2009; Louwerse, 2011; Meteyard et al., 2012; Kiefer and Barsalou, 2013), in contrast, propose that concepts are essentially represented in distinct modality-specific areas. These theories postulate that conceptual features are represented through cell assemblies distributed over sensory, motor, introspective and emotional brain regions. In accordance with Hebbian theory (Hebb, 1949), these functional neural networks result from simultaneous activations of already existing local and distributed cell assemblies in modality-specific areas (Pulvermuller and Fadiga, 2010). Hence, conceptual knowledge is highly dependent on individual experience (Kiefer et al., 2007; Beilock et al., 2008; Lyons et al., 2010; Willems et al., 2010; Hoenig et al., 2011). It is assumed that these modality-specific areas functionally contribute to conceptual comprehension (Pulvermüller, 2005). According to recently emerging hybrid models, conceptual knowledge is the result of processing in modality-specific brain circuits, which interact with multimodal connection hubs (Kiefer and Pulvermüller, 2012; Garagnani and Pulvermuller, 2016). The latter are thought to serve general semantic binding and integration.

Studies supporting grounded cognition theories mainly investigated the representation of concrete concepts, like “hammer” or “to ring” (for reviews see: Kiefer and Pulvermüller, 2012; Meteyard et al., 2012; Kiefer and Barsalou, 2013; for an extension of grounded cognition theories to abstract concepts, see below). Several studies using functional neuroimaging techniques showed that processing of action- (e.g., Hoenig et al., 2008), visual- (Simmons et al., 2007), gustatory- (e.g., Barros-Loscertales et al., 2012), olfactory- (Gonzalez et al., 2006), and sound-related (Kiefer et al., 2008) concepts elicits activations in corresponding modality-specific brain regions. Similar results providing evidence in favor of grounded cognition theories derived from electrophysiological (e.g., Trumpp et al., 2014), behavioral (e.g., Garcia and Ibanez, 2016), neuropsychological (e.g., Trumpp et al., 2013) and transcranial magnetic stimulation (TMS) studies (e.g., Pulvermüller et al., 2005).

While the grounding of concrete object concepts in the sensory and motor systems is well documented, the mere existence of abstract concepts such as “beauty”, “freedom” and “justice” is a serious challenge for the grounded cognition framework. Abstract concepts are characterized by a lack of unique physical features, such as form, color and texture and hence lack a clearly perceivable referent (Crystal, 2004). As a clear physical referent, which can be experienced by our senses, is missing (Paivio, 1986), grounded cognition approaches must be extended in order to account for the representations of abstract concepts. To this end, refined grounded cognition approaches to abstract concepts such as the affective embodiment account (AEA; Kousta et al., 2009), the language and situated simulation theory (LASS; Barsalou et al., 2008) and the words as social tools approach (WAT; Borghi and Binkofski, 2014) have been developed (for similiar approaches, also see Louwerse, 2011; Connell and Lynott, 2012, 2014). They emphasize the importance of linguistic (Barsalou et al., 2008; Kousta et al., 2009; Borghi and Binkofski, 2014), social (Borghi and Binkofski, 2014), introspective (Kiefer and Barsalou, 2013), and affective (Kousta et al., 2009) experiential information for the representation of abstract concepts, in addition to sensorimotor information (see Connell, 2018) – which is utilized through metaphoric relations to concrete concepts as stated in the conceptual metaphor theory (Lakoff and Johnson, 1980). The significance of social, affective and introspective information can be illustrated when considering the concepts “freedom” and “justice”. Thinking about freedom might simulate experiential information one gathered within the hippie movement in the 1960s, feeling free and maybe a bit rebellious (affective and introspective aspects) while protesting with like-minded people for a social upheaval (social aspect). Thinking about justice on the other hand might evoke information about a court hearing with two opposing parties (e.g., hippies vs. policemen; social aspect), where the winning party gets into the flush of victory while the losing party feels shocked and ruined (affective and introspective aspects).

Based on these considerations, a clear-cut distinction between concrete and abstract concepts, as suggested for instance by Paivio (1986) in his Dual Code Theory, is at least questionable (see also Wiemer-Hastings et al., 2001; Connell and Lynott, 2012): Paivio proposed that abstract concepts are stored in a verbal-symbolic code only, whereas concrete concepts rely on both a visual imaginary and a verbal-symbolic code. In line with Paivio’s reasoning, it has been claimed more recently that abstract concepts require amodal, verbal-symbolic representations (Mahon and Caramazza, 2009).

However, this notion of a strict dichotomy between concrete and abstract concepts with abstract concepts relying only on verbal-symbolic representations has been challenged (Wiemer-Hastings and Xu, 2005). Abstract concepts frequently activated left hemispheric language regions in neuroimaging studies (Desai et al., 2011; Sakreida et al., 2013) suggesting a relatively higher importance of verbal associations for this conceptual category. However, activity within the sensorimotor system was also obtained for both abstract and concrete words (Pexman et al., 2007), indicating that abstract concepts also depend on sensory and motor information.

Other research also suggested that concrete and abstract concepts rather differ with regard to their situational content than their representation format (Wiemer-Hastings and Xu, 2005). Although a bimodal distribution of concreteness ratings indicated a categorization of abstract and concrete concepts into two large clusters, there was also a large variance within these clusters (Wiemer-Hastings et al., 2001). Furthermore, introspection-based aspects determined abstractness ratings. The role of internal states for the meaning of abstract concepts was confirmed in a further property listing study by Barsalou and Wiemer-Hastings (2005). Participants of their study were asked to generate associations with regard to abstract (e.g., “freedom”), concrete (e.g., “car”) and intermediate (e.g., “farm”) concepts. Concrete concepts evoked properties related to objects, locations and behaviors in situations, whereas abstract concepts were mainly associated with introspections, mental states and social aspects of situations with intermediate concepts lying in between. More recent studies using ratings and subsequent hierarchical cluster analyses for a large set of concrete and abstract words also found abstract concepts to be more strongly associated with emotions, social cognition, internal and mental states than concrete concepts (Troche et al., 2014, 2017; Binder et al., 2016). The involvement of introspection and internal states in the processing of abstract concepts is also indicated by neuroimaging work. For instance, Wilson-Mendenhall et al. (2013) showed their participants the abstract concept “convince” during a concept-scene matching task, where brain areas underlying mental states and social interaction became active.

In addition to introspections, social aspects and verbal associations, the semantic content of abstract concepts also seems to depend on the sensory and motor systems, similar to concrete object concepts, albeit perhaps to a somewhat smaller extent (Barsalou and Wiemer-Hastings, 2005; Troche et al., 2014, 2017; Binder et al., 2016). Studies providing modality ratings for concrete and abstract concepts showed an association of sensory experience with all concepts, regardless of whether they are classed as abstract or concrete (Lynott and Connell, 2009, 2013; van Dantzig et al., 2011). Behavioral studies examining the “Action-Sentence-Compatibility Effect” (Glenberg and Kaschak, 2002; Glenberg et al., 2008) indicated a contribution of the motor system to the comprehension of abstract concepts, when participants processed them within sentences. Neuroimaging studies also demonstrated the involvement of sensory and motor brain systems during the processing of abstract emotion words similar to face- and arm-related action words (Moseley et al., 2012; see also Dreyer et al., 2015; Vukovic et al., 2017) or during the processing of physical concepts (e.g., “frequency”) similar to performing rhythmic movements (Mason and Just, 2016).

Taken together, behavioral and neuroimaging studies indicated that abstract concepts not only depend on the verbal system (Wang et al., 2010), but also on a variety of modal systems involving perception, action, emotion and internal states. Although many studies considered abstract concepts as an undifferentiated conceptual category, defined solely by a lack of a perceivable physical referent and contrasted them as a uniform class to concrete concepts (see Wang et al., 2010), the semantic content of abstract concepts might be much richer and highly heterogeneous (Wiemer-Hastings et al., 2001). For instance, differential patterns of conceptual relations for abstract emotional and non-emotional concepts were found (Barca et al., 2017). This might explain, why neuroimaging studies reviewed above revealed inconsistent brain areas implicated in the processing of abstract concepts.

Given the probable heterogeneity of abstract concepts, in the present study, we characterized the semantic content of a large set of 296 abstract concepts using property listings. We determined the relative contribution of modal sensory, motor, introspective or social properties to the semantic content of abstract concepts, in addition to verbal associations. The present work had two goals: (i) Although our property listing study does not formally allow to test competing theories of the representation of abstract concepts, the results of our study are nevertheless informative: The presence of modal properties in participants’ listings would be an important prerequisite for the validity of grounded cognition theories. (ii) The obtained property listings for a large set of abstract concepts should provide an estimate of their semantic feature composition. The property listings provide information with regard to the heterogeneity of feature types across concepts and allows to determine possible subcategories. Results are provided as **Supplementary Material** (see **Supplementary Dataset S1**), which might thus guide future behavioral and neuroimaging work investigating the representation of abstract concepts.

To assess the semantic content of abstract concepts, we used a property generation task similar to Barsalou and Wiemer-Hastings (2005). Participants were asked to write down properties such as features, situations and associations coming into mind for 296 abstract concepts. These properties were categorized by a coding-scheme making a classification into modality-specific and verbal contents – namely into sensorimotor features, features describing social constellations, internal states and emotions as well as verbal associations – possible. These 296 concepts were furthermore rated with regard to their familiarity and concreteness/abstractness in two ratings. Lemma frequency and word length were also part of the analysis. Hierarchical cluster analyses were used to shed light into the heterogeneity of abstract concepts. In line with the aforementioned theories of the grounded cognition framework, we hypothesized that abstract concepts are grounded in various modal systems, as it already has been shown with regard to social, affective and introspective experiential information. We furthermore expected that abstract concepts are grounded in the sensorimotor system as well, indicated by a considerable percentage of generated sensorimotor properties in the property generation task. Finally, we assumed that abstract concepts can be divided in several subcategories depending on conceptual feature relevance similar to concrete ones.

## Materials and Methods

### Participants

Sixty healthy volunteers (*M*_age_ = 22.4 years, range = 18 – 46 years, 44 females) from Ulm University participated in the property generation task. All participants were native German speakers (two participants grew up bilingually) with no history of psychiatric or neurological disorders. Another 30 healthy, native German speakers – who did not participate in the property generation task – took part in two ratings: 15 of these subjects (*M*_age_ = 24.6 years, range = 19 – 49 years, 10 females) participated in the first rating, another 15 subjects (*M*_age_ = 27.0 years, range = 23 – 32 years, 10 females) took part in the second rating. Subjects gave written informed consent and were paid eight Euro for participation in the property generation task and study credits for taking part in the rating studies, respectively. The procedure of the study was approved by the Ethical Committee of Ulm University.

### Stimuli

Three-hundred word stimuli were selected from a German dictionary (Scholze-Stubenrecht, 2009) on the basis of the operational definition of abstract concepts (Paivio, 1986; Crystal, 2004): Abstract concepts do not relate to entities that can be directly experienced by our senses and hence lack a clearly perceivable referent. To avoid any effects attributable to the word category, we decided to select abstract nouns only. Words, which were too concrete and hardly used in common parlance as well as foreign words, religious concepts and scientific concepts, were not included. To avoid excessive work load in the property generation task, the 300 abstract words were randomly assigned to one of six questionnaires, comprising 50 words each. To further avoid sequence effects, pages within the six questionnaires were randomized five times. Hence, properties for each abstract word were generated by 10 subjects. Four of the 300 words were excluded subsequently, because three of them (“Pech” – “bad luck”/”pitch”, “Vorstellung” – “imagination”/“show”, “Einstellung” – “attitude”/“setting”) turned out to be ambiguous and one of them (“Lachen” – “laugh”) proved to be too concrete. Therefore, a total of 296 abstract words were included in the analyses.

### Procedure

#### Property Generation Task

Participants were informed that the study investigates word generation. The instruction was kept as open as possible in order not to direct answers in any direction. Subjects were asked to generate and write down associations, properties or situations that come into their mind when thinking about the presented words as spontaneously as possible, but without any temporal limitations. Participants were further instructed to write down about four properties and to avoid synonyms for the respective terms. If no property/situation came into mind, participants were told to skip this particular word. Within the instruction, subjects were given two words (e.g., “hallucination”) and potential properties (e.g., “colorful”, “loud”, “hearing voices”) as examples, which were not part of the actual word stimuli.

#### Ratings

Participants were asked to rate 300 abstract and 77 concrete words with regard to familiarity and concreteness/abstractness (valence and arousal ratings were also obtained. However, they were considered for use in future studies and hence were not analyzed here). Familiarity was rated on a 6-point Likert scale on basis of the two poles “low familiarity” and “high familiarity”, with higher scores indicating higher familiarity. The instructions asked the subjects to make their decision based on whether they often use, see or hear the named concepts, or whether the term is rather rarely encountered. A similar scale with the poles “abstract” and “concrete” was used regarding concreteness/abstractness, with higher scores indicating higher concreteness. Subjects were guided by the classical definition of abstractness whereby named concepts with a lack of perceivable physical features should receive ratings of high abstractness (i.e., low scores of concreteness), while terms referring to perceivable objects, persons or materials should be rated with high concreteness scores. The written instructions provided subjects with three examples and potential ratings each (e.g., “joy”, high familiarity, high abstractness; not used in the critical ratings). Subjects were instructed to rate the words as spontaneously as possible.

Ratings were obtained in two separate samples in order to decrease the number of ratings in each subject. In the first sample, participants rated 190 abstract and 49 concrete words on a paper-pencil questionnaire. In the second sample, the remaining 110 abstract as well as 28 concrete words were rated. For better practicability an online questionnaire was used^1^. Concrete words (e.g., “table”, “donkey”) were included in the questionnaires in order to avoid response bias (ratio of abstract to concrete words in both rating studies was approximately equal [∼ 1:4]).

### Data Analysis

#### Data Coding Scheme

A coding scheme adopted from Barsalou and Wiemer-Hastings (2005) was developed in order to qualitatively analyze the generated properties and to assign these properties to one of five main categories. The categories and their definitions are as follows:

(I) *Sensorimotor feature*: A feature that can be experienced by our senses. It describes the meaning of the abstract concept, or the abstract concept can be applied to this feature. In order to investigate the modality-specific nature of abstract concepts more closely, the category “sensorimotor feature” was further divided into seven subcategories: visual (e.g., “colorful painting” for “creativity”), acoustic (e.g., “loud” for “argument”), motor-related (e.g., “hug” for “sympathy”), tactile (e.g., “fluffy” for “comfort”), olfactory (e.g., “sulfurous” for “disgust”), gustatory (e.g., “bitter” for “disgust”) and interoceptive (e.g., “stomach ache” for “hunger”) feature.
(II) *Social constellation*: a feature or a situation that describes the coexistence of different persons or which implies an interaction between at least two different persons, e.g., “friends” for “sympathy”.
(III) *Internal state and emotion*: a feature or a situation that reflects internal, cognitive processes (e.g., motivation, emotion, volition). Also a feature or a situation that describes the character of an individual and which implies an evaluation of the respective abstract concept, e.g., “joy” for “sympathy”.
(IV) *Association*: a feature or a situation that does not describe the abstract concept, but which is thematically or symbolically related to it. This feature does not directly contribute to the understanding of the abstract concept, e.g., “sun” for “sympathy”.
(V) *Other abstract concept*: an abstract feature that describes the abstract concept or to which the abstract concept can be applied. This category also includes all terms that are identical to one of the other words used in the questionnaire, e.g., “karma” for “sympathy”.

Hence, the subjects’ responses were classified into eleven categories. Double coding was possible (e.g., “to paint” as visual and motor-related feature of “talent”).

Two independent coders used the aforementioned coding scheme to classify the generated properties. Coders were trained to achieve high reliability and to keep inter-individual variance as low as possible (see Barsalou and Wiemer-Hastings, 2005; Barca et al., 2017; Trumpp and Kiefer, 2018 for similiar coding procedures). The two coders were different from the authors and naïve to the purpose of the study. They saw the abstract word while coding each property in order to decide whether the generated feature reflects a verbal association or a semantic property of the respective concept. Inter-rater reliability (see Barsalou and Wiemer-Hastings, 2005 for a similar method of reliability analysis) in terms of joint probability of agreement was 76.79 %.

#### Statistical Analysis

Data was analyzed using RStudio (version 1.0.153; RStudio-Team, 2015) and R’s (R-Core-Team, 2017) packages “ez” (Lawrence, 2016), “car” (Fox and Weisberg, 2011), “cluster” (Maechler et al., 2017) and “NbClust” (Charrad et al., 2014). After coding was completed, relative frequencies were calculated for each feature type per concept within each subject (e.g., a participant reported four properties for a specific concept: two motor-related features, one visual feature and one association. Thus, the relative frequency for motor-related features was 2/4 = 0.5, for visual related features and associations 1/4 = 0.25, each). In a second step, relative frequencies for each feature type and concept were averaged across all subjects.

In order to identify significant differences of relative frequencies of generated features between categories, univariate repeated measures analyses of variance were carried out. Level of significance was defined as *p* < 0.05. When significant variation between categories was indicated, *post hoc* testing (Bonferroni *post hoc* tests) was performed. Since we assumed (on the basis of the aforementioned theories) that both categories “association” and “other abstract concept” reflect verbal associations, these categories were combined into the superordinate category “verbal association”. The first analysis considered the category “sensorimotor feature” as a whole and thus consisted of the four factor levels “sensorimotor feature”, “social constellation”, “internal state/emotion” and “verbal association”. To further investigate the distribution of the sensorimotor features in detail, a second analysis was carried out in which the seven specific sensorimotor features were compared.

Welch’s *t*-test was used to compare concreteness/abstractness and familiarity ratings of concrete and abstract concepts. This analysis also had the purpose of validating the selection of our stimuli by showing that our selected abstract concepts were indeed rated abstract. Welch’s *t*-test was chosen because of the different number of stimuli per word category. Levene’s test also indicated that the assumption of homogeneity of variances was not fulfilled.

Additional correlation (Pearson’s *r*) analyses were performed to examine possible relationships between concreteness/abstractness ratings and the generated characteristics, the ratings of familiarity and eventually lemma frequency (derived from the German lexical database dlexDB; Heister et al., 2011) and word length (number of letters). Subsequent regression analyses with concreteness/abstractness ratings as the dependent variable aimed at answering the questions of what constitutes abstractness and of how the observed heterogeneity within the concreteness/abstractness ratings can be explained.

To further identify possible homogeneous subgroups of abstract concepts with distinct profiles of generated features, hierarchical cluster analyses were conducted (see **Supplementary Data S2, S3** for the R script of our cluster analyses and the matching data set). Hierarchical cluster analyses were used in order to generate internally homogeneous clusters while variability between clusters is maximized. Cluster Analysis 1 was based on the four clustering variables “sensorimotor feature”, “social constellation”, “internal state/emotion” and “verbal association”. Cluster Analysis 2 was based on the seven specific sensorimotor features (e.g., visual, motor-related, …) in order to investigate the structure of the data in a more detailed fashion. Cluster analyses were carried out on the basis of Euclidean distances as a measure of distances between clusters. To identify and subsequently remove outliers, the single-linkage clustering method was used. The single-linkage method, which is based on minimal distances between clusters and tends to produce chaining effects, is highly sensitive to the presence of outliers, since objects with extreme differences to all other objects are the last ones to converge. Visual inspection of the resulting dendrogram can consequently be used as a tool to remove outliers (Steinbach et al., 2004). After eliminating the outliers, Ward’s (1963) method, which minimizes within-group dispersion based on a sum-of-squares criterion (Murtagh and Legendre, 2014), was applied to the data. Because there is no universally accepted method for determining the optimal number of clusters, we took into account theory-driven, visual (inspection of the dendrograms, and the “elbow test”) and statistical (based on the “NbClust” package in R 3.4.1.: a package providing 26 indices, such as Calinski and Harabasz index and Silhouette index; Charrad et al., 2014) criteria.

We did not conduct an additional factor analysis because we were interested in finding different subcategories of abstract concepts characterized by a multidimensional feature space and not in reducing the number of underlying feature dimensions.

## Results

On average, 3.37 properties per word were generated showing that participants followed the instructions.

### Analysis 1 – Overview

**FIGURE 1:**
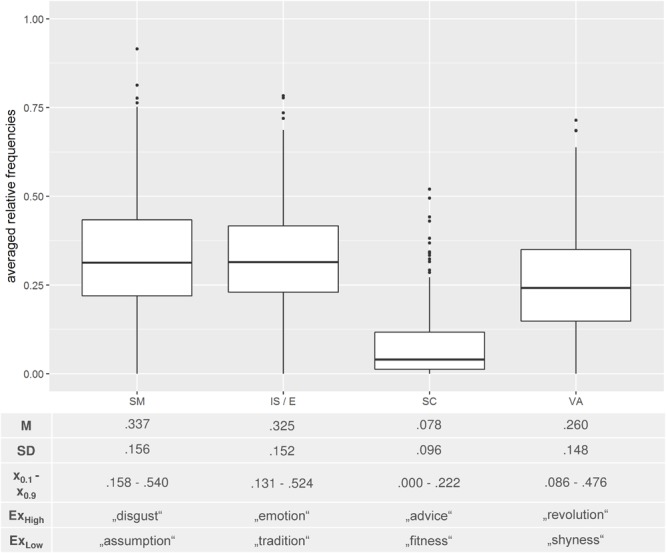
Descriptive statistics of Analysis 1 and corresponding boxplots. *M* = mean; *SD* = standard deviation; *x_0.1/0.9_* = first and ninth decile. SM = sensorimotor feature; IS/E = internal state/emotion; SC = social constellation; VA = verbal association. Ex_High_/Ex_Low_ depicts exemplary abstract concepts with a high/low portion of generated features in the respective categories.

The first analysis comprised four categories: “sensorimotor feature” (SM), “social constellation” (SC), “internal state/emotion” (IS/E) and “verbal association” (VA). **Figure 1** shows relevant descriptive statistics and the corresponding boxplots. At the descriptive level, participants generated the highest portion of features within the “sensorimotor feature” category (*M* = 0.337) followed by the “internal state/emotion” (*M* = 0.325), the “verbal association” (*M* = 0.260) and the “social constellation” (*M* = 0.078) categories. A univariate repeated measures ANOVA revealed that relative frequency of generated features differed significantly between categories [*F*(3,885) = 161.19, *p* < 0.001; Greenhouse–Geisser-corrected *p*-value]. *Post hoc* comparison using Bonferroni tests revealed significant differences between all conditions (all *p*s < 0.001).

As can be seen in the boxplots (**Figure 1**), the ranges within categories were quite large, reflecting a rather heterogeneous generation of properties. This becomes particularly evident when considering the “sensorimotor feature” category, where subjects generated between 0.0 and 91.6% sensorimotor features per word. A similar result pattern was also observed with regard to the other categories (range_internal state/emotion_ = 0.00 – 0.783, range_social constellation_ = 0.00 – 0.521, range_verbal association_ = 0.00 – 0.715). **Figure 1** (lower part) gives an overview of examples reflecting a rather high or a rather low portion of generated features in the respective categories.

### Analysis 2 – Distribution of Sensorimotor Features in Detail

**FIGURE 2:**
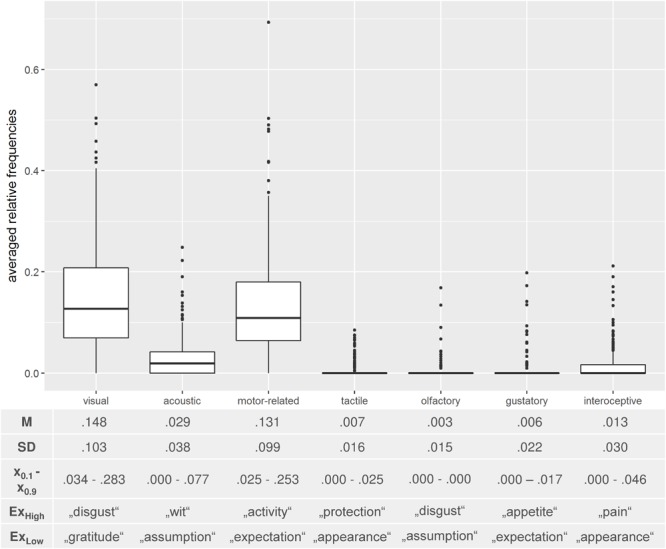
Descriptive statistics of Analysis 2 and corresponding boxplots. *M* = mean; *SD* = standard deviation; *x_0.1/0.9_* = first and ninth decile. Ex_High_/Ex_Low_ depicts exemplary abstract concepts with a high/low portion of generated features in the respective categories.

The second analysis comprised the seven modality-specific subcategories “visual”, “acoustic”, “motor-related”, “tactile”, “olfactory”, “gustatory” and “interoceptive”. Descriptive statistics and corresponding boxplots are shown in **Figure 2**. At the descriptive level the highest relative frequencies were generated within the categories “visual” (*M* = 0.148) and “motor-related” (*M* = 0.131), followed by the acoustic category (*M* = 0.029). All other categories (*M_tactile_* = 0.007, *M_olfactory_* = 0.003, *M_gustatory_* = 0.006, *M*_interoceptive_ = 0.013) only played a marginal role in the generation of properties. Relative frequencies differed significantly between the subcategories as shown by a second univariate repeated measures ANOVA [*F*(6,1770) = 346.34, *p* < 0.001; Greenhouse–Geisser-corrected *p*-value]. *Post hoc* Bonferroni tests revealed that relative frequency did not differ between the “visual” and “motor-related” categories (*p* = 1.00) and between the “tactile”, “gustatory” and “olfactory” categories (all *p*s > 0.10). All other comparisons were statistically significant (all *p*s < 0.05).

Inspection of the boxplots in **Figure 2** also indicates that ranges within single categories were relatively large. Especially the categories “visual”, “motor-related” and “acoustic” showed a broad range (range_visual_ = 0.00 – 0.569, range_motor-related_ = 0.00 – 0.693, range_acoustic_ = 0.00 – 0.248). Examples reflecting a rather high or a rather low portion of generated features in these categories are shown in the lower part of **Figure 2**.

### Comparison Between Concrete and Abstract Concepts

In order to validate that our selected abstract concepts were indeed abstract, abstract and concrete concepts were compared with regard to concreteness/abstractness ratings. Data from concrete concepts were obtained from two ratings, in which concrete concepts served as fillers. In these rating studies, in addition to concreteness/abstractness, ratings of familiarity were also available. Welch’s *t*-test revealed that concreteness/abstractness ratings (for relevant descriptive statistics see **Table 1**) differed significantly between the two concept classes [*t*(83.2) = -51, *p* < 0.001]. Abstract concepts (*M* = 2.55; *SD* = 0.53; range = 1.33 – 4.27) were rated significantly more abstract than concrete concepts (*M* = 5.72; *SD* = 0.38; range = 3.80 – 6.00). When looking at the quantiles, it is noticeable that concreteness/abstractness ratings were much more heterogeneous within abstract concepts than in concrete concepts. Considering concrete concepts, 90% of the values were lying between 5.40 and 5.95, while 90% of the ratings regarding abstract concepts were lying within a much broader window between 1.93 and 3.27. Taking this into account, it is not surprising that the abstract concept class yielded some isolated outliers ($\bar{x}$ ± 2σ) with regard to their concreteness/abstractness ratings. Concepts like “thirst” (*M* = 4.27), “work” (*M* = 4.13) and “assassination” (*M* = 4.07) were rated relatively concrete, whereas concepts like “fantasy” (*M* = 1.33), “honor” (*M* = 1.40) and “miracle” (*M* = 1.40) were rated rather abstract.

**Table 1:** Descriptive statistics of concreteness/abstractness and familiarity ratings for abstract and concrete concepts.

		*M*	*SD*	*Min*	*Max*	*x_0.1_*	*x_0.5_*	*x_0.9_*
Concreteness	Abstract concepts	2.552	0.532	1.333	4.267	1.933	2.533	3.267
	Concrete concepts	5.722	0.378	3.800	6.000	5.400	5.800	5.947
Familiarity	Abstract concepts	4.463	0.732	2.467	5.800	3.400	4.533	5.467
	Concrete concepts	4.392	0.855	1.733	5.800	3.480	4.400	5.293

*M = mean; *SD* = standard deviation; *Min* = minimum; *Max* = maximum; *x_0.1/0.9_* = first and ninth decile; *x_0.5_* = median.*

While concreteness/abstractness ratings were quite diverging, familiarity ratings showed no significant differences [*t*(60.2) = 0.551, *p* = 0.584]. Abstract concepts were rated with *M* = 4.46 (*SD* = 0.73; range = 2.47 – 5.80) on average, concrete concepts were rated with a mean rating of *M* = 4.39 (*SD* = 0.86; range = 1.73 – 5.80).

### Correlation and Regression Analyses

In order to explain the observed range within the abstract concept class and to investigate possible relationships between concreteness/abstractness ratings and other variables, correlation analyses were carried out. Significant correlations within the superordinate categories were observed between concreteness/abstractness ratings and relative frequency of the “sensorimotor feature” and “internal state/emotion” categories, familiarity ratings and lemma frequency, respectively (**Table 2A**; all *p*s < 0.01).

**Table 2A:** Results of correlation analyses for abstract concepts with the superordinate categories.

	SM	IS/E	SC	VA	Familiarity	Word length	Lemma frequency
Concreteness	0.397^∗∗∗^	–0.354^∗∗∗^	0.058	–.092	0.346^∗∗∗^	–0.026	0.175^∗∗^

*SM = sensorimotor feature; IS/E = internal state/emotion; SC = social constellation; VA = verbal association. ^∗^*p* < 0.05; ^∗∗^*p* < 0.01; ^∗∗∗^*p* < 0.001.*

Within the seven modality-specific subcategories, significant positive correlations between concreteness/abstractness ratings and relative frequency of the “visual”, “motor-related”, “olfactory”, “gustatory” and “interoceptive” feature categories were found (**Table 2B**; all *p*s < 0.05).

**Table 2B:** Results of correlation analyses for abstract concepts with the modality-specific subcategories.

	Vis	Acou	Mot	Tac	Olf	Gus	Int
Concreteness	0.218^∗ ∗ ∗^	0.103	0.208^∗ ∗ ∗^	0.082	0.126^∗^	0.240^∗ ∗ ∗^	0.203^∗ ∗ ∗^

*vis = visual; acou = acoustic; mot = motor-related; tac = tactile; olf = olfactory; gus = gustatory; int = interoceptive. ^∗^*p* < 0.05; ^∗∗^*p* < 0.01; ^∗∗∗^*p* < 0.001.*

In order to examine the direction of the relationship more precisely, a first multiple linear regression analysis was conducted – on the basis of the aforementioned correlation analysis – to predict concreteness/abstractness ratings based on relative frequency of the “sensorimotor feature” and “internal state/emotion” superordinate categories, familiarity ratings and lemma frequency (**Table 3A**). A significant regression equation was found [*F*(4,291) = 34.3, *p* < 0.001], with an adjusted *R^2^* of 0.311. Relative frequency of the categories “sensorimotor feature” [*β* = 0.280, *t*(291) = 4.97, *p* < 0.001] and “internal state/emotion” [*β* = -0.228, *t*(291) = -4.04, *p* < 0.001] as well as familiarity ratings [*β* = 0.330, *t*(291) = 6.49, *p* < 0.001] were identified as significant predictors of concreteness/abstractness ratings. Relative frequency of the “sensorimotor feature” category and familiarity ratings had significant positive regression weights, indicating words with higher scores on these scales were expected to be rated more concrete, after controlling for the other variables in the model. Relative frequency of the “internal state/emotion” category had a significant negative weight, indicating that after accounting for the other variables, those concepts with a higher portion of “internal state/emotion” features were expected to be rated more abstract. Lemma frequency did not account for a significant portion of the variance after controlling for the other variables (*p* = 0.115).

**Table 3A:** Results of multiple linear regression analysis for abstract concepts with concreteness/abstractness as dependent variable and relative frequency of the “sensorimotor feature” and “internal state/emotion” categories, familiarity ratings and lemma frequency as predictors.

*R^2^_adj_.* = 0.311 [*F*(4,291) = 34.3, *p* < 0.001]			
	Coefficient estimate (*β*)	***SE***	***t***
Sensorimotor feature	0.280^∗∗∗^	0.056	4.97
Internal state/Emotion	–0.228^∗∗∗^	0.057	–4.04
Familiarity	0.330^∗∗∗^	0.051	6.49
Lemma frequency	0.081	0.051	1.58

*All variables in the model have been tested for collinearity by using variance inflation factors (VIFs). All VIFs were < 5.0 indicating that the independent variables are not collinear (Urban and Mayerl, 2006); *R^2^_adj_*_._ = adjusted *R^2^*; *β* = standardized coefficient; *SE* = standard error, *t* =*

Similarly, a second multiple linear regression analysis, in which the superordinate category “sensorimotor feature” was replaced as predictor by the more fine-grained subcategories “visual”, “motor-related”, “olfactory”, “gustatory” and “interoceptive”, was conducted to predict concreteness/abstractness ratings (**Table 3B**). Again, a significant regression equation was found [*F*(8,287) = 18.2, *p* < 0.001], with an adjusted *R^2^* of 0.318. Relative frequency of the categories “motor-related” [*β* = 0.164, *t*(287) = 3.20, *p* < 0.01], “gustatory” [*β* = 0.141, *t*(287) = 2.17, *p* < 0.05], “interoceptive” [*β* = 0.158, *t*(287) = 2.99, *p* < 0.01] and “internal state/emotion” [*β* = -0.265, *t*(287) = -4.44, *p* < 0.001] as well as familiarity ratings [*β* = 0.322, *t*(287) = 6.26, *p* < 0.001] were identified as significant predictors of concreteness/abstractness ratings. Visual features as predictor for concreteness/abstractness just failed to reach significance (*p* = 0.051). Relative frequency of the “motor-related”, “gustatory” “interoceptive” and “visual” subcategories as well as familiarity ratings had significant positive regression weights, indicating words with higher scores on these scales were expected to be rated more concrete. Again, relative frequency of the “internal state/emotion” category had a significant negative weight, indicating that those concepts with a higher portion of “internal state/emotion” features were expected to be rated more abstract. Lemma frequency, here too, did not account for a significant portion of the variance (*p* = 0.093).

**Table 3B:** Results of multiple linear regression analysis for abstract concepts with concreteness/abstractness as dependent variable and relative frequency of the “visual”, “motor-related”, “olfactory”, “gustatory”, “interoceptive” and “internal state/emotion” categories, familiarity ratings and lemma frequency as predictors.

*R^2^_adj_.* = 0.318 [*F*(8,287) = 18.2, *p* < 0.001]			
	Coefficient estimate (*β*)	*SE*	*T*
Visual	0.114	0.058	1.96
Motor-related	0.164^∗∗^	0.051	3.20
Olfactory	–0.072	0.061	–1.17
Gustatory	0.141^∗^	0.065	2.17
Interoceptive	0.158^∗∗^	0.053	2.99
Internal state/Emotion	–0.265^∗∗∗^	0.060	–4.44
Familiarity	0.322^∗∗∗^	0.051	6.26
Lemma frequency	0.087	0.052	1.68

*All variables in the model have been tested for collinearity by using variance inflation factors (VIFs). All VIFs were < 5.0 indicating that the independent variables are not collinear (Urban and Mayerl, 2006); *R^2^_adj_*_._ = adjusted *R^2^*; *β* = standardized coefficient; *SE* = standard error, *t* = t-value. ^∗^*p* < 0.05; ^∗∗^*p* < 0.01; ^∗∗∗^*p* < 0.001.*

### Hierarchical Cluster Analyses

#### Cluster Analysis 1

**FIGURE 3:**
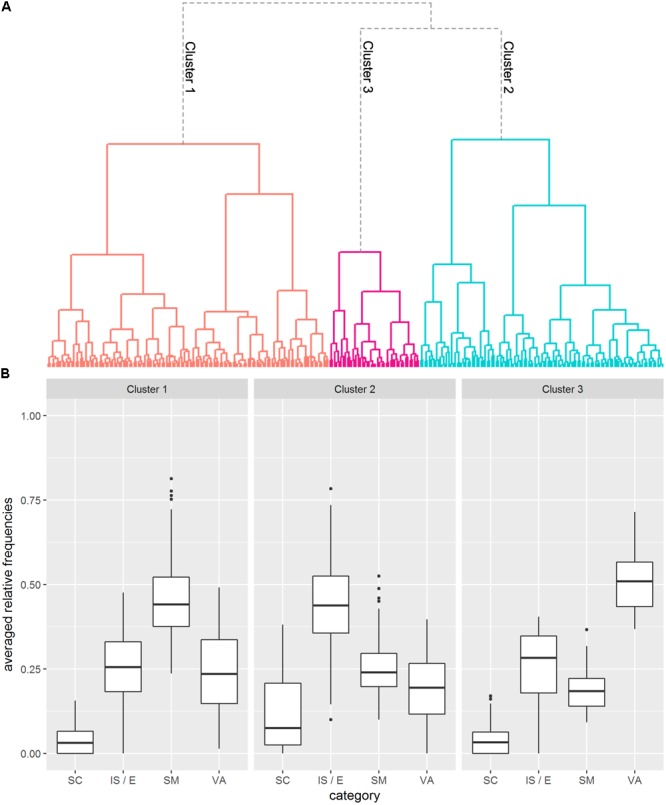
Results of Cluster Analysis 1. **(A)** Dendrogram visualizing the *k* = 3 cluster solution. The different clusters are marked by different colors. **(B)** Boxplots depicting generated features per cluster (SM = sensorimotor feature; IS/E = internal state/emotion; SC = social constellation; VA = verbal association).

Cluster Analysis 1 was based on the four clustering variables “sensorimotor feature”, “social constellation”, “internal state/emotion” and “verbal association”. The single-linkage clustering method led to the exclusion of ten outliers. **Figure 3A** shows the dendrogram based on the subsequent cluster analysis with Ward’s method. The structure of the dendrogram as well as the “elbow test” (**Supplementary Figure S1**) indicated that the optimal number of clusters is *k* = 3. In addition, most of the criteria of the “NbClust” function (7 of 26; across *k* = 2 and *k* = 5 clusters) spoke in favor of a three cluster solution.

**Figure 3B** shows boxplots of generated features per cluster. Cluster 1, the largest cluster (*n* = 131), was characterized by a relatively high portion of sensorimotor features (*M* = 0.459, *CI_0.95_* = [0.438;0.480]). Features in the “social constellation” category played only a subordinate role in this cluster (*M* = 0.041, *CI_0.95_* = [0.034;0.049]), while properties of both the “internal state/emotion” (*M* = 0.257; *CI_0.95_* = [0.239;0.275]) and the “verbal association” (*M* = 0.243; *CI_0.95_* = [0.223;0.263]) categories were generated to an intermediate extent. Abstract concepts in this cluster are for example “observation”, “insight” and “fitness”.

Specific for Cluster 2, which contains *n* = 113 abstract concepts, was a large portion of generated features of the “internal state/emotion” category (*M* = 0.437; *CI_0.95_* = [0.411;0.463]). Besides that, compared to the other two clusters, this cluster had more than twice as many properties generated in the “social constellation” category (*M* = 0.115; *CI_0.95_* = [0.095;0.134]). While sensorimotor features (*M* = 0.255; *CI_0.95_* = [0.240;0.271]) were generated at a medium level, verbal associations (*M* = 0.193; *CI_0.95_* = [0.176;0.210]) played a marginal role. Typical examples in this cluster are “nightmare”, “argument” and “criticism”.

The situation is quite different with Cluster 3, the smallest cluster (*n* = 42) in which verbal associations were clearly in the foreground (*M* = 0.508; *CI_0.95_* = [0.479;0.537]). Like in Cluster 1, features in the “social constellation” category were subordinate (*M* = 0.049; *CI_0.95_* = [0.033;0.065]). Although the categories “sensorimotor feature” (*M* = 0.188; *CI_0.95_* = [0.167;0.209]) and “internal state/emotion” (*M* = 0.255; *CI_0.95_* = [0.220;0.290]) were generated at an intermediate level, they play the least important role here compared to the other clusters. “Present”, “theory” and “dignity” are examples in this cluster.

#### Cluster Analysis 2

**FIGURE 4:**
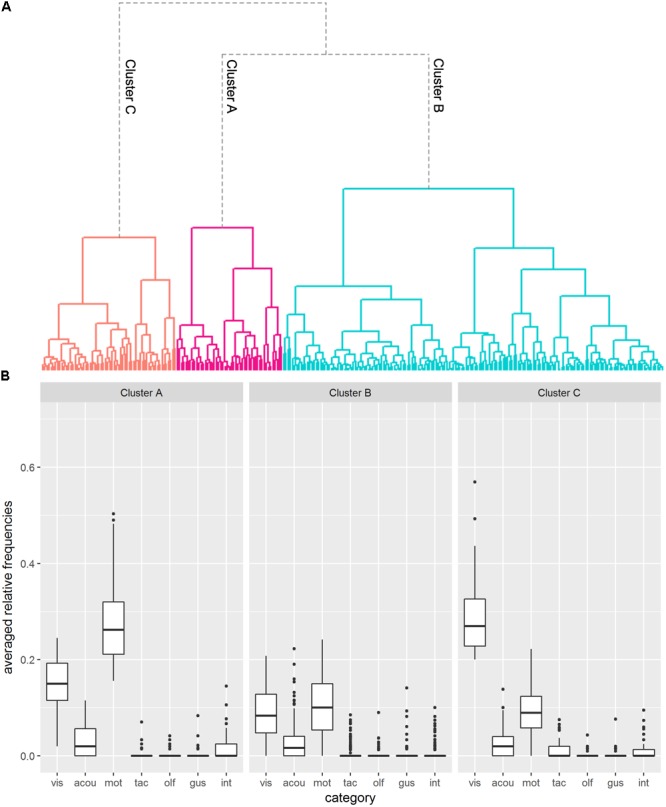
Results of Cluster Analysis 2. **(A)** Dendrogram visualizing the *k* = 3 cluster solution. The different clusters are marked by different colors. **(B)** Boxplots depicting generated features per cluster (vis = visual; acou = acoustic; mot = motor-related; tac = tactile; olf = olfactory; gus = gustatory; int = interoceptive).

Cluster Analysis 2 comprised the seven modality-specific subcategories “visual”, “acoustic”, “motor-related”, “tactile”, “olfactory”, “gustatory” and “interoceptive”. Ten outliers were identified by the single linkage clustering method. The remaining 286 abstract concepts were subsequently analyzed using Ward’s method (see **Figure 4A** for the resulting dendrogram). The structure of the dendrogram, the “elbow test” (**Supplementary Figure S2**) as well as most of the criteria of the “NbClust” function (15 of 26; across *k* = 2 and *k* = 5 clusters) spoke in favor of a *k* = 3 cluster solution.

Inspection of **Figure 4B** reveals that the three clusters hardly differ with regard to acoustic, tactile, olfactory, gustatory and interoceptive features. These categories played a rather subordinate role in the clusters. Considering that these features were generated less frequently in general (see point “Analysis 2”), this is not very surprising. Categories distinguishing the clusters were the “visual” and “motor-related” categories.

Cluster A, which was the smallest cluster with *n* = 49 abstract concepts, was characterized by a large proportion of motor-related features (*M* = 0.279; *CI_0.95_* = [0.254;0.305]), the largest proportion across the three clusters. Visual features were less frequently generated (*M* = 0.146; *CI_0.95_* = [0.128;0.163]). Typical examples in this cluster are “fitness”, “fight” and “performance”.

For Cluster B, by far the biggest cluster (*n* = 175), visual (*M* = 0.092; *CI_0.95_* = [0.084;0.100]) and motor-related (*M* = 0.102; *CI_0.95_* = [0.093;0.111]) features were approximately equally generated, albeit at an intermediate level. Abstract concepts in this cluster are for example “experience”, “challenge” and “humor”.

Feature structure of Cluster C (*n* = 62) almost looked like a mirror-inverted version of Cluster A. It was characterized by the highest portion of generated features in the “visual” category compared to the other clusters (*M* = 0.289; *CI_0.95_* = [0.270;0.308]). Features of the “motor-related” category (*M* = 0.092; *CI_0.95_* = [0.078;0.107]), on the other hand, were generated at an intermediate level. “Observation”, “vanity” and “beauty” are examples in this cluster.

## Discussion

The present study investigated the semantic content of 296 abstract concepts using a property generation task similar to Barsalou and Wiemer-Hastings (2005). The quintessence of our results is that abstract concepts are heterogeneous, as shown by our descriptive and cluster analyses. Our study therefore extends previous work on the semantic content of abstract concepts (Lynott and Connell, 2009, 2013; van Dantzig et al., 2011; Troche et al., 2014, 2017; Binder et al., 2016) by showing that different clusters of abstract concepts can be distinguished according to their specific semantic featural composition.

Participants generated a considerable amount of properties in all feature categories. The distribution of the observed properties fits well into the picture of previous work investigating the representation of abstract concepts. Our results suggest that social (Carpenter et al., 1998; Bergelson and Swingley, 2013; Borghi et al., 2017) and emotional/introspective features (Wiemer-Hastings et al., 2001; Kousta et al., 2011; Vigliocco et al., 2014) as well as verbal associations (Simmons et al., 2008; Wang et al., 2010; Recchia and Jones, 2012) play a crucial role in the processing of abstract concepts as demonstrated in previous behavioral, developmental, brain imaging and patient studies (Kiefer and Pulvermüller, 2012; Meteyard et al., 2012; Borghi et al., 2017). Similarly, recent rating studies targeting the semantic content of concrete and abstract concepts also indicated the specific relevance of social, emotional and introspective features for constituting the semantic content of abstract concepts (Troche et al., 2014, 2017; Binder et al., 2016). In terms of quantity, sensorimotor features played the most important role in our study. This is quite remarkable, given that sensorimotor features are thought to be only associated with concrete concepts, but not with abstract concepts (Wang et al., 2010). More recent studies, however, indicate the significance of the sensory and motor system even in the representation of abstract concepts (Lynott and Connell, 2009, 2013; Moseley et al., 2012; Troche et al., 2014, 2017; Dreyer et al., 2015; Binder et al., 2016; Mason and Just, 2016; Vukovic et al., 2017). The importance of sensorimotor features becomes evident when thinking, for example, about the abstract concept “beauty”, where, at least in the western society, visual properties seem to play a pivotal role in conceptual representation.

The cluster analyses furthermore demonstrated that the class of abstract concepts is characterized by a multidimensional feature space with differential dominant properties (Troche et al., 2014, 2017) as outlined in recent theories on conceptual cognition (Binder et al., 2016). Similar to concrete concepts, abstract concepts can be divided into different subcategories according to the dominance of certain conceptual features, although the number of subcategories appears to be more limited, probably because some features are less dominant in abstract concepts (e.g., olfactory or gustatory features). The subcategories found with help of Cluster Analysis 1 correspond to refined grounded cognition theories, which emphasize the role of social, emotional and introspective features as well as verbal associations: Cluster 2, which was characterized by high proportions of generated features in the “internal state/emotion” and “social constellation” categories, is compatible with assumptions made by Barsalou and Wiemer-Hastings (2005); Vigliocco et al. (2014), and Borghi and Binkofski (2014), all theories underlining the importance of emotional, introspective and/or social features in the representation of abstract concepts. Cluster 3, in which features of the “verbal association” category were dominant, can also be reconciled with theories such as WAT (Borghi and Binkofski, 2014) and LASS (Barsalou et al., 2008), which highlight the role of linguistic experiential information. Beyond that, Cluster 1 showed that sensory and motor-related features also play a crucial role in the representation of abstract concepts. Cluster Analysis 2 showed that, within the sensorimotor feature category, visual and motor-related features are closely related (Jeannerod, 1999), as it already has been reported in the case of concrete concepts (Tyler and Moss, 2001). Since we have not conducted a similar hierarchical cluster analysis with concrete concepts, the comparison of abstract and concrete concepts and possible resulting clusters must remain speculative. Based on previous results (Barsalou and Wiemer-Hastings, 2005; Wang et al., 2010; Troche et al., 2014, 2017; Trumpp and Kiefer, 2018), it is likely that the cluster characteristics of abstract and concrete concepts would have differed. Clusters within the concrete concept class might be characterized by an even higher portion of sensorimotor features, whereas social, emotional and introspective features as well as verbal associations might play a less important role (Trumpp and Kiefer, 2018). A final answer to this question might be given by future studies directly comparing cluster analyses of abstract and concrete concepts.

Results from the ratings validated the abstractness of the selected abstract words. The selected abstract concepts were indeed rated more abstractly than the concrete concepts, while they were, at the same time, not generally more unfamiliar. Similar to Wiemer-Hastings et al. (2001), we found a bimodal distribution of concreteness ratings forming two almost distinct patterns localized over the concrete and abstract center of the scale, respectively. However, we also observed a wide variance within these patterns, weakening the strict dichotomous view of classical approaches (e.g., Paivio, 1986). Especially the concreteness/abstractness ratings of abstract concepts showed a relatively large variance. This large variance within abstract concepts can partly be explained by their semantic content and their familiarity (see also Troche et al., 2014, 2017; Binder et al., 2016). Within abstract concepts, familiarity ratings were lower the less concrete these concepts were. This might be due to the fact that the most abstract concepts, like “boycott”, play only a subordinate role in everyday life. As a consequence, less frequent use of these concepts could reduce the possibility for situational sensory and motor experiences resulting in ratings of higher abstractness. In accordance with earlier findings of Wiemer-Hastings et al. (2001), but based on a much larger sample of abstract concepts, we found that emotional/introspective features were related to higher abstractness ratings, while higher concreteness ratings were more likely to go hand in hand with a high portion of sensorimotor features and high familiarity ratings (see also Troche et al., 2014, 2017). This differential relation of sensorimotor and emotional/introspective features to concreteness/abstractness becomes particularly evident when considering the two abstract concepts “expectation” (emotional/introspective) and “fitness” (sensorimotor). While thinking about the word meaning of “expectation” is accompanied by a myriad of possible contexts and situational components, the less abstract entity “fitness” is much more stronger linked to specific context information rendering it relatively more concrete (see also Schwanenflugel and Shoben, 1983; Schwanenflugel et al., 1992; Schwanenflugel and Akin, 1994). When considering the different sensorimotor features separately, motor-related, gustatory and interoceptive features were significantly related to concreteness. The association between visual features and concreteness just failed to reach the conventional significance level (*p* = 0.051). Note, however, that several concerns have been raised regarding traditional approaches of gathering concreteness/abstractness ratings. Connell and Lynott (2012), for example, point out that concreteness/abstractness ratings can be biased by unfavorable instructions, resulting in different decision criteria at the two poles “concrete” and “abstract” of the scale (e.g., excessive focus on vision while neglecting other modalities).

Although the present property listing study does not formally allow to test competing theories of the representation format of abstract concepts, our findings are difficult to reconcile with theories claiming that abstract concepts purely rely on a verbal-symbolic code, like Paivio’s (1986) Dual Code Theory does. If the assumptions of the Dual Code Theory were true, we would have expected a much higher proportion of verbal associations in our study. Although Cluster 3 from Cluster Analysis 1 is compatible with Paivio’s assumption, the remaining two clusters – which are characterized by dominant modal features – clearly oppose it. Additionally, the large variance of generated properties also speaks against Paivio’s (1986) theory, although the frequency of verbal associations might be somewhat underestimated in our property generation task, whose instruction emphasized semantic properties of the concepts as well as associations. The broad diversity in participants’ listings is rather consistent with refined grounded cognition theories by showing that the semantic content of abstract concepts – just like that of concrete concepts – includes introspective, affective, social, sensory and motor-related features. Our results are thus consistent with theories such as LASS (Barsalou et al., 2008), AEA (Kousta et al., 2009), WAT (Borghi and Cimatti, 2009), and Louwerse’s (2011) Symbol Interdependency Hypothesis, which emphasize the importance of linguistic, social, introspective and affective experiential information for the representation of abstract concepts. Our results, of course, do not necessarily imply that the modal feature types found here are also represented in the corresponding modal brain areas as claimed by grounded cognition theories. However, our results constitute an important prerequisite for further tests of the validity of the grounded cognition framework.

Based on the present results we propose for future studies to abandon the traditional approach (Paivio, 1986) of considering abstract concepts as undifferentiated conceptual category and to contrast them with concrete concepts. Instead, we show that abstract concepts have a rich and heterogeneous semantic content with emphasis on different feature categories. We therefore suggest that a comparison of well-specified subcategories of abstract concepts – as revealed by the present cluster analyses – is more appropriate to investigate the processing of abstract concepts at a behavioral or neural level (Wilson-Mendenhall et al., 2012, 2013; Barca et al., 2017). For instance, a comparison of visual- and motor-related abstract concepts might clarify to what extent the motor and visual system is involved in the representation of those abstract concepts. Further imaging studies could shed light on the neural correlates of conceptual processing using the present results for their stimulus selection (see **Supplementary Dataset S1**), while studies with brain lesioned patients and with TMS would allow conclusions, which dominant features are functionally relevant for specific subcategories of abstract concepts.

## Ethics Statement

This study was carried out in accordance with the recommendations of the Ethical Committee of Ulm University with written informed consent from all subjects. All subjects gave written informed consent in accordance with the Declaration of Helsinki. The protocol was approved by the Ethical Committee of Ulm University.

## Author Contributions

MH, NT, and MK planned the study design. NT and MK supervised the study. MH performed the data acquisition, analyzed the data, and wrote the first draft of the paper. MH, MK, and NT revised the manuscript. All the authors approved the final version of the manuscript.

## Conflict of Interest Statement

The authors declare that the research was conducted in the absence of any commercial or financial relationships that could be construed as a potential conflict of interest.

## Footnotes

1 http://www.unipark.com/
