# Supplementary material for: The Semantic Content of Abstract Concepts: A Property Listing Study of 296 Abstract Words
Source: Front Psychol. 2018 Sep 19;9:1748. doi: 10.3389/fpsyg.2018.01748 (PMC6156367; doi:10.3389/fpsyg.2018.01748)
Supplement: Supplementary file 1 [file Table_1.docx]

Supplementary Material

The Semantic Content of Abstract Concepts: A Property Listing Study of 296 Abstract Words

Marcel Harpaintner, Natalie M. Trumpp, Markus Kiefer*

*** Correspondence:** Markus Kiefer: markus.kiefer@uni-ulm.de

# Supplementary Data

**Supplementary Data S1.** Data gained by the property generation task, the ratings and the hierarchical cluster analyses.

**Supplementary Data S2.** R script containing the R code for the hierarchical cluster analyses.

**Supplementary Data S3.** Data used in the R script (Data S2).

# Supplementary Figures


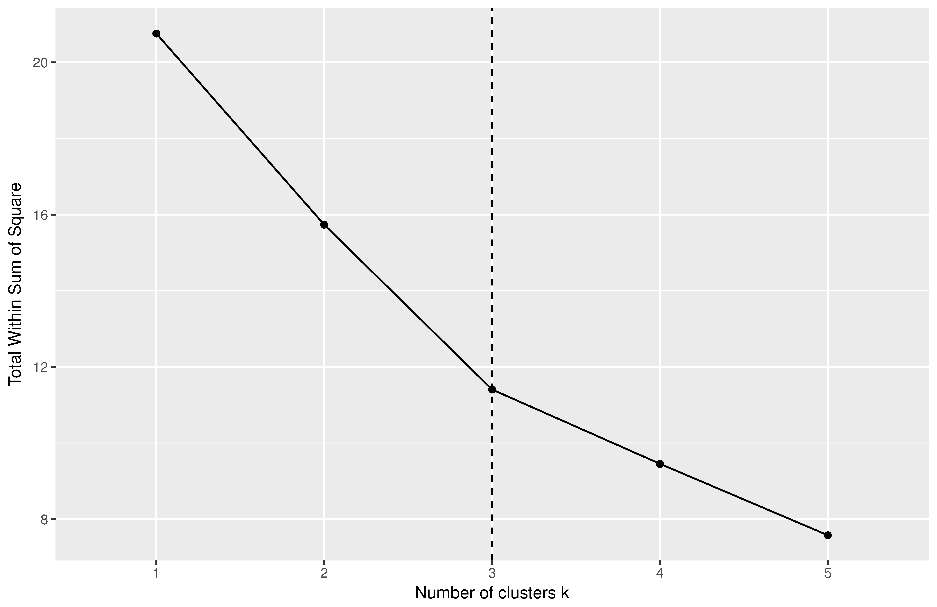


**Supplementary Fig S1.** Hierarchical cluster “elbow test” of Cluster Analysis 1. Total within sum-of-square (WSS) as a function of the number of clusters. The optimal cluster solution is indicated by the point at which adding additional clusters does not substantially reduce the WSS. The vertical dashed line depicts this critical point (k = 3).


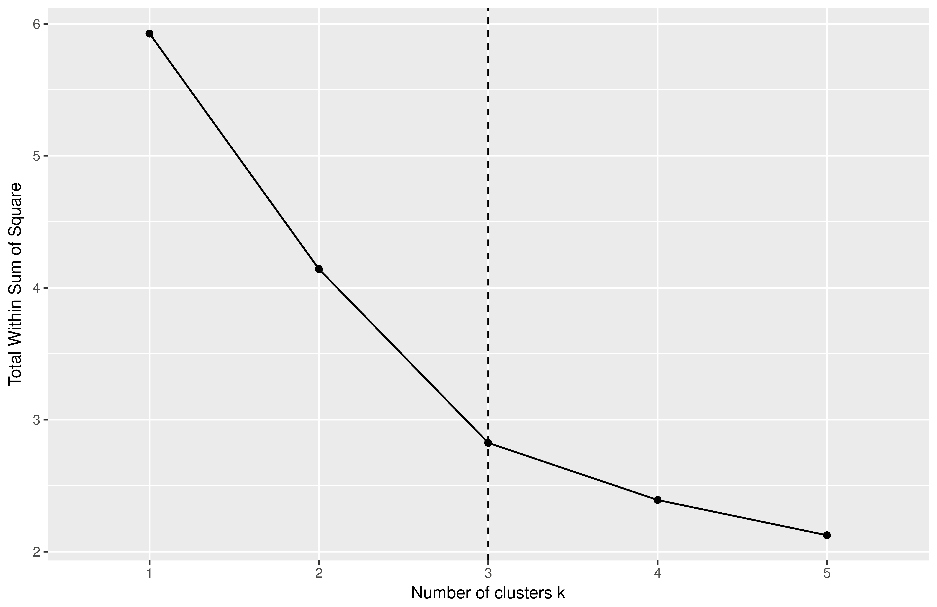


**Supplementary Fig S2.** Hierarchical cluster “elbow test” of Cluster Analysis 2. Total within sum-of-square (WSS) as a function of the number of clusters. The optimal cluster solution is indicated by the point at which adding additional clusters does not substantially reduce the WSS. The vertical dashed line depicts this critical point (k = 3).
